# Multiple tropical Andean glaciations during a period of late Pliocene warmth

**DOI:** 10.1038/srep41878

**Published:** 2017-02-07

**Authors:** Nicholas J. Roberts, René W. Barendregt, John J. Clague

**Affiliations:** 1 Department of Earth Sciences, Simon Fraser University, 8888 University Drive, Burnaby, V5A 1S6, Canada; 2 Department of Geography, University of Lethbridge, 4401 University Drive West, Lethbridge, T1K 6T5, Canada

## Abstract

The extent and behaviour of glaciers during the mid-Piacenzian warm period illustrate the sensitivity of the cryosphere to atmospheric CO_2_ concentrations above pre-industrial levels. Knowledge of glaciation during this period is restricted to globally or regionally averaged records from marine sediments and to sparse terrestrial glacial deposits in mid-to-high latitudes. Here we expand the Pliocene glacial record to the tropics by reporting recurrent large-scale glaciation in the Bolivian Andes based on stratigraphic and paleomagnetic analysis of a 95-m sequence of glacial sediments underlying the 2.74-Ma Chijini Tuff. Paleosols and polarity reversals separate eight glacial diamictons, which we link to cold periods in the benthic oxygen isotope record. The glaciations appear to coincide with the earliest glacial activity at high northern latitudes and with events in Antarctica, including the strong M2 cold peak and terminal Pliocene climate deterioration. This concordance suggests inter-hemispheric climate linkages during the late Pliocene and requires that the Central Andes were at least as high in the late Pliocene as today. Our record fills a critical gap in knowledge of Earth systems during the globally warm mid-Piacenzian and suggests a possible driver of faunal migration preceding the large-scale biotic interchange in the Americas during the earliest Pleistocene.

The late Pliocene (Piacenzian Stage) was a time of global warmth that preceded climatic deterioration of the earliest Pleistocene. At times during the late Pliocene, global mean temperatures were ~2–3 °C warmer than present^1^,^2^,^3^,^4^ and atmospheric CO_2_ concentrations were similar to those of today^5^,^6^. Records of glaciation during the late Pliocene thus provide insight into responses of the Earth system to CO_2_ above pre-industrial levels^4^,^5^,^7^. The mid-Piacenzian (3.265–3.025 Ma^8^) is the most recent of Earth’s warm periods and has, therefore, been a major focus of climate research, providing an opportunity to improve predictive models of near-future climate^7^,^8^,^9^.

Existing late Pliocene glacial records are regional or global averages derived from elemental and isotopic compositions of marine microorganisms^2^,^3^,^10^, ice-rafted detritus in sub-polar seas^11^,^12^, global ice volumes^10^,^13^, sea level^10^,^14^, or atmospheric CO_2_ concentrations^5^,^6^. These records suggest several expansions of glacier ice during the late Pliocene, followed by gradual step-wise cooling near the end of the epoch.

Well dated terrestrial glacial deposits improve paleoclimate reconstructions by directly recording local conditions^12^,^15^. Prior to the Pleistocene, however, nearly all indisputable terrestrial glacial deposits are restricted to middle to high latitudes (>45°) and even these records are sparse^12^. Cenozoic glacial records from the tropics are almost completely limited to the Quaternary, particularly the Holocene^16^ and latest Pleistocene^17^,^18^.

The only direct evidence of Pliocene tropical glaciation comes from the Bolivian Andes, at the base of a ~500-m-thick glacial sequence exposed along the edge of the Altiplano just north of La Paz^12^,^15^ (Fig. 1). The oldest exposed glacial sediments, first reported by Dobrovolny^19^, are 7 m of oxidized till (Patapatani Drift) directly below a dacitic tuff (Chijini Tuff) ~7 km north of the city centre (PTE in Fig. 1b). He suggested an Early Pleistocene age for this till based on mammalian fossils found within what he interpreted to be coeval glaciofluvial outwash 30 m below the Chijini Tuff ~12 km farther south. Later researchers^20^,^21^ argued against the existence of glacial sediments below the Chijini Tuff and suggested that till overlying the tuff (Calvario Drift) records the earliest glaciation of the Cordillera Real to the northeast. Clapperton^22^ assigned the Patapatani Drift to the late Pliocene based on a biotite K-Ar age of 3.27 ± 0.14 Ma on the tuff at a nearby section where it overlies 3 m of till (Fig. 1b). Subsequent researchers^23^,^24^ have argued that biotite K-Ar ages in this region are too old due to mineral alteration (Supplementary Information). Their K-Ar^23^ and ^40^Ar/^39^Ar^24^ ages on potassium feldspar in the Chijini Tuff, which range from 2.650 ± 0.012 to 2.8 ± 0.1 Ma, are considered to be reliable and confirm that underlying sediments are pre-Pleistocene.

We document nearly 100-m-thick sequences of glacial diamictons separated by paleosols below the Chijini Tuff in new road cuts (Figs 1c and 2, and Supplementary Figure S1) in the Río Kaluyo valley near Dobrovny’s^19^ type section (Fig. 1b). This section is informally referred to as the Patapatani West section. We studied the stratigraphy and sedimentology of the glacial sequence, determined the paleomagnetic polarity of its fine-grained sediments, and obtained a new ^40^Ar/^39^Ar age on sanidine from the Chijini Tuff.

## Lithostratigraphy and magnetostratigraphy

A 2-m-thick, poorly sorted, clast-supported, pebble-cobble gravel (unit 1) with some striated clasts marks the base of the exposed sequence (Fig. 3a). It is overlain by 6 m of stratified, matrix-supported diamicton (unit 2) with common faceted and striated clasts (Supplementary Figure S2) that are preferentially oriented in a NE-SW direction (Supplementary Figure S3). The diamicton contains discontinuous, decimeter-thick mud lenses (Fig. 3a). Clasts in units 1 and 2 consist mainly of argillite and phyllite. These are the dominant bedrock lithologies exposed in the Andes to the northeast^25^.

Unit 2 is unconformably overlain by 87 m of massive to very weakly stratified, matrix-supported diamicton with 20–30 percent clasts up to boulder size, a muddy matrix, and rare silt and sand lenses (Figs 2 and 3a). Many clasts are striated and faceted (Supplementary Figure S2). Conspicuous boulder-cobble lags are present within the sequence (Fig. 2). Four reddened horizons, each characterized by a combination of columnar to blocky structure, clay skins, magnetite enrichment, upward fining, and a sharp upper contact (Supplementary Figure S2 and Supplementary Table S1), divide the sequence into five diamicton units (units 3–7). The Chijini Tuff (unit 8) comprises 20–45 cm of loose friable ash at its base, transitioning upward into ~9.5 m of cliff-forming, weakly cemented ash. A massive diamicton overlies the tuff and is similar to diamicton units below it (Fig. 3a and Supplementary Figure S2 and S3). The percentage of granitic clasts increases from ~50% in units 3–5 to ~90% in units 6 and 7, presumably reflecting progressive unroofing of Triassic and Jurassic granitic plutons in the Cordillera Real^25^. All sub-tuff diamictons (units 3–7) have strong unimodal clast fabrics with orientations ranging from NNE-SSW to NW-SE (Supplementary Figure S3).

We consider unit 1 to be proglacial outwash. Unit 2 may be the deposit of a glaciogenic debris flow, suggestive of a more ice-proximal setting. Oxidized zones capping diamicton units 3, 4, 5, and 6 are paleosols, indicating subaerially exposed surfaces that were stable for thousands to tens of thousands of years (Supplementary Information). Common striated and faceted clasts show that matrix-supported, generally massive, diamicton units below the tuff (units 3–7) are glacial in origin. They were deposited either beneath or at the margin of glaciers during separate glaciations. Strong clast alignment is consistent with high subglacial shear stresses^26^,^27^, suggesting ice flow initially to the south-southwest and later to the south-southeast (Supplementary Figure S3). Alternatively, it is possible that the diamictons were deposited at the margins of glaciers on aprons bordering the subsiding La Paz basin. We acknowledge some uncertainty in the details of the depositional environment, but either interpretation requires that glaciers extended at least 14 km from the high Cordillera Real. In light of the long periods of landscape stability indicated by each of the four paleosols, these glaciers formed during at least five separate glaciations. The presence of the undisturbed tuff throughout La Paz area^19^,^23^,^24^ indicates emplacement on a subaerial surface, suggesting an interglacial eruption.

Paleomagnetic remanence measurements (Fig. 4; Supplementary Information) indicate that the sampled materials are stably magnetized and that cleaned directions record a primary remanence representing true paleofield directions. Some units record a secondary component of magnetization of opposite polarity to that of the primary component (Supplementary Figure S4), indicating partial remagnetization following a post-depositional reversal of the geomagnetic field. Unit mean directions for both normal and reversed polarities are near the expected Geocentric Axial Dipole (GAD) field position (Fig. 4d; Supplementary Table S2). The sequence records four polarity reversals (Figs 3c and 4d; Supplementary Table S1), and thus represents a considerable length of geologic time. Two polarity reversals within unit 3 indicate that it comprises three subunits (3a, 3b, 3c: Fig. 3a,c), possibly separated by hiatuses.

## Glacial chronology and expansion

The new ^40^Ar/^39^Ar age on sanidine −2.74 ± 0.04 Ma (Supplementary Figure S5) – confirms that the Chijini Tuff is latest Pliocene in age and allows polarity data to be confidently correlated to the geological timescale (Fig. 3e). The polarity sequence constrains all diamicton units within the Gauss Chron (Fig. 3e), which spans the period 3.588–2.608 Ma (late Pliocene). Two polarity zones in the sequence (normally magnetized mid-Gauss Chron, C2An.2n; and reversely magnetized Kaena subchron, C2An.2r) are completely within the mid-Piacenzian warm period. The oldest normal magnetozone in the section (N1 in Fig. 3d) is likely early Gauss and not an older subchron within the Gilbert Chron, as the latter would require a major hiatus in the sediment sequence, spanning much of the 0.596-Ma^13^ reversely magnetized C2Ar subchron (latest Gilbert Chron).

Reliable correlation of terrestrial glacial deposits with specific marine isotope stages (MIS) is rarely possible^28^, especially prior to the Late Pleistocene. However, the short duration of late Pliocene polarity intervals before 3.045 Ma and the predominance of a single cold peak during most of these intervals enables us to assign some glacial events in the section to specific cold periods of the global, astronomically tuned, benthic oxygen isotope (δ^18^O) record^13^ (Fig. 3f). The strongest and longest cold peaks are most likely to generate far-reaching Andean glaciers. Long hiatuses in the sequence, such as those recorded by paleosols, can be similarly linked to the warmest and longest interglacials. Near-linear fits between the geomagnetic polarity timescale and magnetostratigraphy of fine-grained sediments of late Pliocene age in the central La Paz basin^21^ suggest continuous, long-term aggradation of the fill sequence beneath the Altiplano. Hiatuses in the described sections therefore probably represent periods of non-deposition rather than erosion that might remove evidence of earlier events.

Of the nine pre-Chijini glacial units defined by polarity reversals and unconformities, six correspond unambiguously to five specific marine isotope stages: MG2, M2, KM2, G22 and G10 (solid diamonds in Fig. 3f). Two glacial units (3a and 3b) record the single strong cold peak within their respective subchrons (M2 during C2An.2r and KM2 during C2An.2n). Formation of a paleosol between the two tills that fall within the Kaena subchron (units 3c and 4) requires glaciation during both of that subchron’s strong cold peaks (KM2 and G22, respectively); MIS KM2 is thus recorded by two tills of opposite polarity, reflecting its occurrence at a polarity reversal. Units 2 and 7 are most likely to have been deposited during the strongest and latest of multiple cool peaks (MG2 and G10, respectively) preceding the start of the Mammoth subchron and deposition of the Chijini Tuff. Each of the other three glacial units is constrained to a small number of similar-magnitude cold peaks (open diamonds in Fig. 3f) during a given polarity subchron.

In this interpretation, units 1–3 record either three or four glaciations, depending on whether unit 1 was deposited shortly before unit 2 during MIS MG2 or during an earlier glaciation in MIS MG1 (Fig. 3f). The presence of well-developed paleosols, marking interglacial conditions, between units 4–7 indicates that these tills record four subsequent glaciations. The measured sequence below the tuff thus records either seven or eight glaciations. The expansion of Andean ice indicated by the shift from a proglacial environment (unit 1) to an ice-marginal or subglacial environment (unit 3a) records climate deterioration directly preceding and during the globally recognized^12^,^29^ MIS M2 (ca. 3.3 Ma) cooling event. An Andean ice cap formed again during each of the two coolest parts of the mid-Piacenzian warm period (KM2 and G22) and in three subsequent glaciations (ending with G20) during climatic deterioration between ca. 3.0–2.8 Ma.

**Figure 1:**
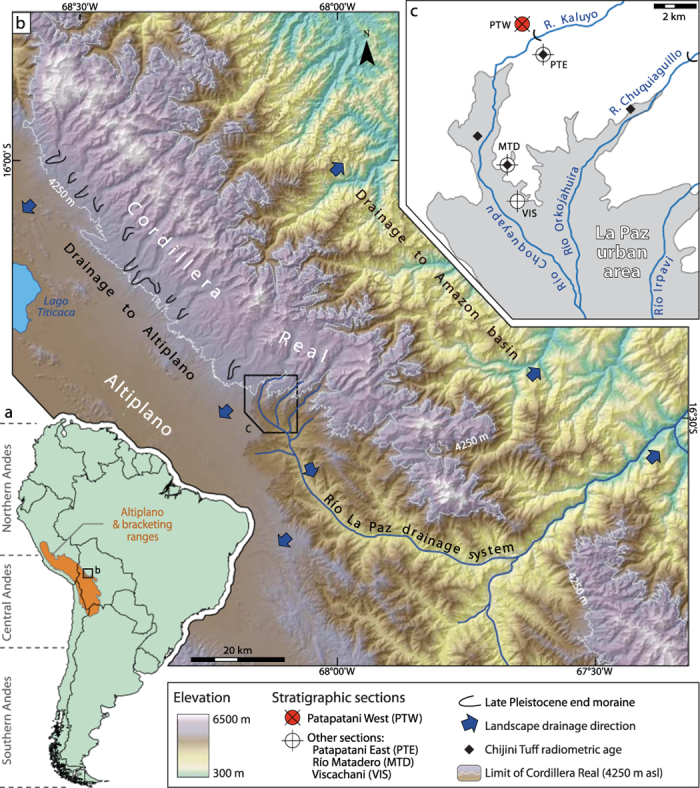
Geographic and physiographic context of the Cordillera Real. (**a**) Setting of the Central Andes within South America. (**b**) Physiography of the Cordillera Real in the vicinity of La Paz. Terrain is from the ASTER GDEM 2 produced by METI and NASA. (**c**) Overview of the northern La Paz basin showing locations of the Patapatani West section and other sections described by previous authors: PTE – Patapatani East section^19^; MTD – Río Matadero section^22^; and VIS – Viscachani section^20^,^21^. See Supplementary Information for details of radiometric ages on the Chijini Tuff. Maps created using ArcGIS 9.3 and Adobe Illustrator CS3.

**Figure 2:**
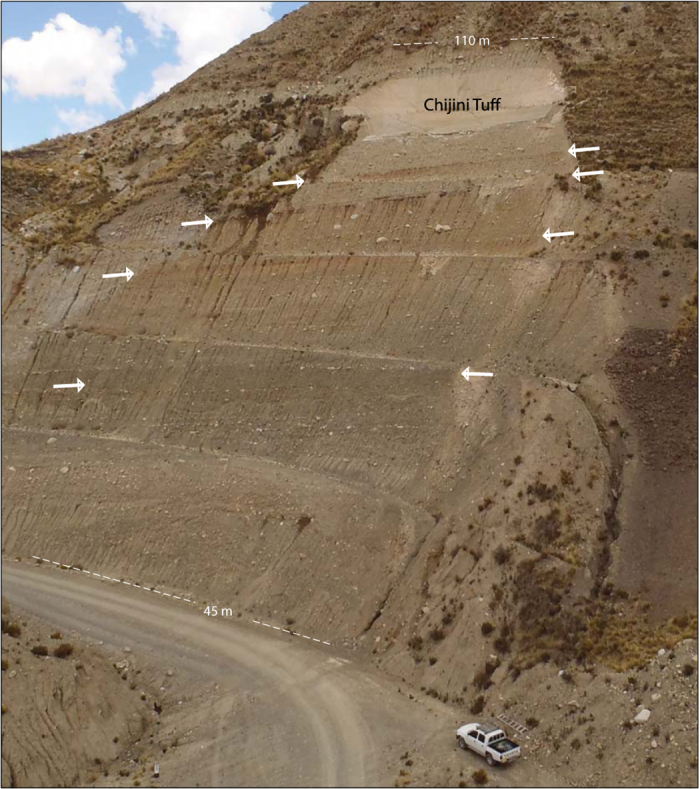
Upper part of the Patapatani West section. The road cut exposes 10 m of Chijini Tuff capping 50 m of massive to weakly stratified diamicton containing four buried soils (paired white arrows). The exposure shown here spans the zone from 45 m to 110 m in Fig. 3. See Supplementary Figure S1 for an overview of the entire section and a detailed view of the lower part of the stratigraphic sequence.

Magnetostratigraphic correlation of the Patapatani West section to the Viscachani section^21^, 6.5 km farther south (Figs 1b and 5), corroborates our chronostratigraphic interpretation and identifies a shift in late Pliocene depositional environments away from the Cordillera Real. Agreement in the number and thickness of magnetozones at the two sections makes it unlikely that any subchrons of the Gauss Chron were missed during sampling. Similar average sediment accumulation rates for the pre-Chijini glacial sequence at the Patapatani West section (15 cm/ka) and the Gauss-aged upper La Paz Formation underlying the tuff at the Viscachani section (16 cm/ka) are within the range of Plio-Pleistocene basin aggradation rates elsewhere in the Central Andes (9 to 17 cm/ka; Supplementary Information and Supplementary Table S3). The silt-dominated sequence at Viscachani indicates reduced transport energy compared to the Patapatani West section. The first gravel beds, 75 m below the tuff, at the Viscachani section and the increase in the number of sand beds in the overlying sequence suggest a general increase in transport energy that is roughly coincident with the facies change from proglacial to glacial conditions at the Patapatani West section (Fig. 5). Thus, parts of the fine-grained fluvial and lacustrine sediments of the upper La Paz Formation underlying the Chijini Tuff are distal outwash associated with Pliocene glaciers to the north, as postulated by Dobrovolny^19^.

The potential for preserving evidence of Pliocene glaciations in high-mountain environments is extremely low due to erosion during subsequent, more extensive Pleistocene glaciations^12^. The long, relatively continuous, late Pliocene glacial sequence north of La Paz is the fortuitous result of deposition in a subsiding basin during the Pliocene and subsequent incision of the Altiplano during the Pleistocene. Repeated glaciation of the Cordillera Real following uplift during the Miocene^30^,^31^ led to large-scale sediment transfer into the subsiding basin to the west.

Glacial sediment units exposed at our study site undoubtedly continue along the western flank of the high Cordillera Real northwest of La Paz and possibly also along lower parts of the Cordillera Real to the southeast, but they are buried beneath thick accumulations of Pleistocene drift. Their position at the Patapatani West section is similar to those of Late Pleistocene terminal moraines^18^,^19^,^32^ (Fig. 1b,c). Pliocene glacial deposits are unknown on the eastern flank of the Cordillera Real, likely having been eroded during the Pleistocene. If moisture was ultimately derived from the equatorial Atlantic, as during the Late Pleistocene, late Pliocene glaciers probably descended low on these slopes in the headwaters of the Amazon Basin. Late Pliocene ice caps may thus have had widths of the order of 30–40 km over a distance of at least 80 km along the axis of the Cordillera Real, comparable in extent to the Last Glacial Maximum ice cap^18^,^32^ (Fig. 1).

## Discussion

**Figure 3:**
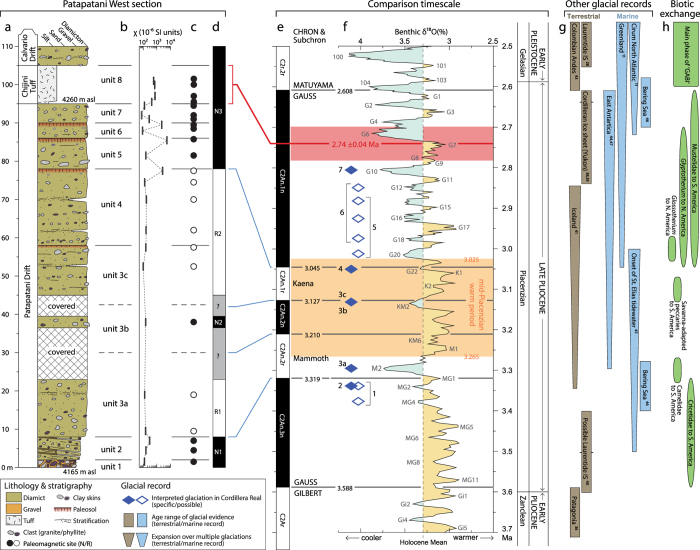
Lithostratigraphy and magnetostratigraphy of the Patapatani West section, correlation with the geomagnetic polarity time scale and oxygen isotope record, and comparison with contemporary glacial records. (**a**) Lithostratigraphy. (**b**) Mean magnetic susceptibility by sample group. (**c**) Polarity by sample group. (**d**) Interpreted polarity sequence. (**e**) Astronomically tuned geomagnetic polarity time scale^13^. (**f**) Benthic δ^18^O paleo-temperature profile^13^ (prefixes of Pliocene stages reflect the geomagnetic subchron in which they occur^13^) and timing of Cordillera Real glaciations. (**g**) Other early Pliocene to earliest Pleistocene glacial records (Kleiven *et al*.^11^; Balco *et al*.^28^; Mercer^36^; Barendregt *et al*.^38^; Hidy *et al*.^39^; Gao *et al*.^40^; Geirsdóttir^41^; Helmens *et al*.^43^; Passchier^44^; Lagoe *et al*.^45^; Takahashi *et al*.^46^; McKay *et al*.^47^). (**h**) Single-taxa, inter-American land mammal exchanges leading up to the Great American Biotic Interchange (GABI) (details in Supplementary Information).

Differing interpretations of the time of Cenozoic uplift of the Central Andes^30^,^33^ result in uncertainties in their elevation during the Pliocene and consequently on their range of influence on Southern Hemisphere atmospheric circulation^7^,^30^. Similarity of our inferred late Pliocene glacial limits with Late Pleistocene end moraines^18^,^19^ suggests that the Cordillera Real was at least as high in the late Pliocene as it is today. Arguably, the Pliocene Cordillera Real was even higher than today, because ice extent during the less intense late Pliocene cold peaks was similar to that of the stronger oxygen isotope excursion of the Last Glacial Maximum^13^ (slightly more than 4‰ versus ~5‰) (Fig. 3f). Till overlying the Chijini Tuff, which is of latest Pliocene or earliest Pleistocene age, extends ~10 km beyond the Patapatani sections^19^,^20^, indicating that the first post-2.74 Ma ice caps were more extensive than those reported here. However, these inferences do not consider possible differences in moisture and heat availability in the Central Andes in the late Pliocene that might be expected due to changes in ocean and atmosphere circulation^1^,^2^,^34^ and that are important to contemporary mass balance of tropical glaciers^35^.

The fragmentary record^12^ and limited chronological resolution^28^ of most Pliocene terrestrial glacial deposits (Fig. 3g) hinder their correlation with the Central Andean sequence described in this paper. The Patagonian records include a till tightly constrained to the latest Gilbert Chron (3.71–3.588 Ma) based on magnetic polarity and K-A chronology^36^, as well as a till overlying a 3.46-Ma basalt flow^36^ and outwash gravel overlain by a basalt flow of about the same age as the Chijini Tuff^37^. The latter two glacial deposits may fall within the period reported here. The earliest reliably dated terrestrial evidence of ice sheet glaciation in North America – till^38^ and outwash^38^,^39^ in central Yukon Territory – dates to the end of the Pliocene, about 2.64 Ma^38^,^39^, more than 700,000 years after the first evidence of glaciation at La Paz. A possible late Pliocene till in James Bay Lowland, Canada (~53°N) is constrained only by the polarity sequence (top down R-N-R-N) of overlying lacustrine sediments and imprecise age control based on pollen biostratigraphy^40^. There are questions about the reliability of the interpretation, but if correct the purported till either correlates with or is older than the deposit of the earliest glaciation described here. No direct evidence of Pliocene glaciation has yet been found in Europe, Scandinavia, or Greenland^12^. Late Pliocene terrestrial glacial deposits in Iceland record local glaciations in the Kaena subchron and preceding early Gauss, as well as two regionally extensive, highland ice caps in the late Gauss after ca. 2.9 Ma^41^. Outside the Cordillera Real, the earliest known evidence of tropical Cenozoic glaciation is on Mount Kenya where glacial diamictons date to the Olduvai subchron and earlier Matuyama Chron (between 2.068 and 1.781 Ma)^42^ and in the Andes of Colombia where glaciofluvial sediments have been reported from the earliest Pleistocene (ca. 2.6 Ma)^43^.

**Figure 4:**
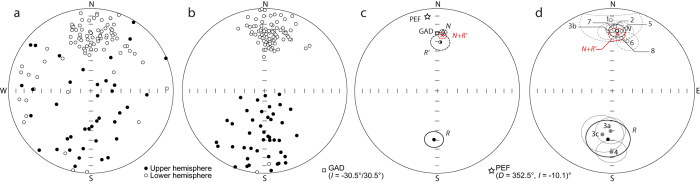
Paleomagnetic remanence directions. Ellipses are 95% confidence limits. (**a**) NRM directions for all samples. (**b**) Cleaned (ChRM) directions for all samples. (**c**) Mean of cleaned directions for all samples (*N*, normally magnetized samples; *R*, reversely magnetized samples; *R*′, transposed reversely magnetized samples; *N* + *R*′ [red ‘X’], all samples irrespective of polarity projected on upper hemisphere; GAD, normally magnetized Geocentric Axial Dipole; PEF, Earth’s present magnetic field. (**d**) Mean of cleaned directions by stratigraphic unit and means of unit means (*N*, normally magnetized units; *R*, reversely magnetized units; *N* + *R*′ [red ‘X’], all units irrespective of polarity projected on upper hemisphere).

**Figure 5:**
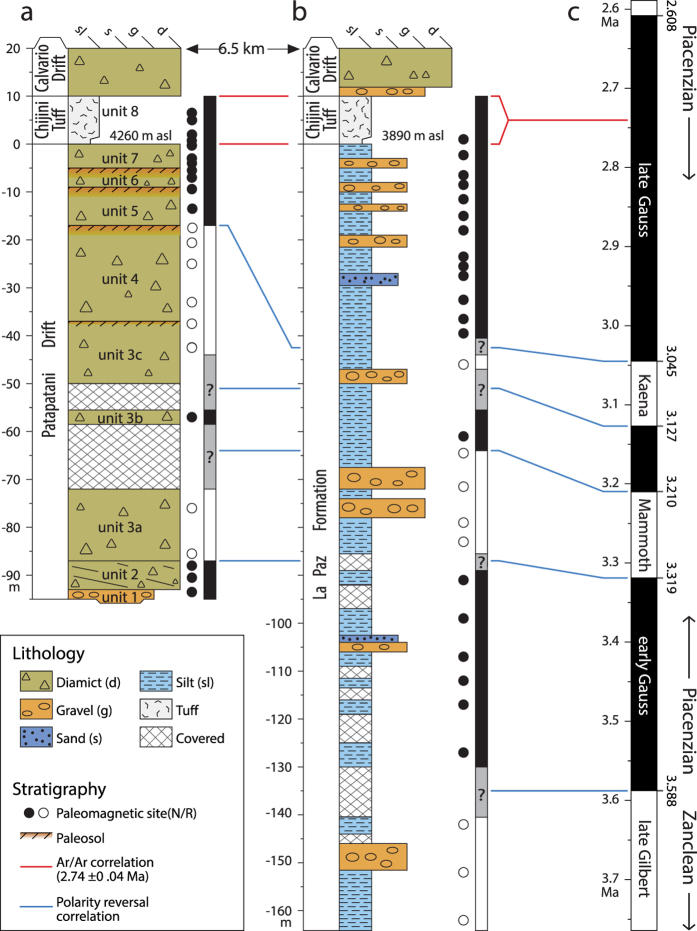
Correlation of the Patapatani West section with the Viscachani section showing distal-fining away from the Cordillera Real. Note the similarity of polarity sequences at the two sites. (**a**) The Patapatani West section (see Fig. 3 for detail). (**b**) The Viscachani section, which was covered by urban development in the late twentieth century. Previous studies provide its lithostratigraphy^20^ and magnetostratigraphy^21^. (**c**) Polarity sequence for the end of the early Pliocene (late Gilbert Chron) and the late Pliocene (Gauss Chron). The ages of polarity reversals are from the astronomically tuned LR04 geomagnetic polarity time scale^13^.

More reliable correlations with pre-Chijini glaciations of the Cordillera Real are possible from continuous marine sequences recording the onset of high-latitude tidewater glaciation (Fig. 3g), leading up to and during the major IRD increase in the circum-North Atlantic at ca. 2.7 Ma (MIS G6)^12^. Calving of ice from Greenland^11^ and East Antarctica^44^ increased slightly during MIS MG2, when unit 2 and possibly unit 1 were deposited north of La Paz. Polar ice rafting then increased substantially during MIS M2^11^,^44^, prior to the mid-Piacenzian warm period and coincident with deposition of unit 3a. Deposition of glaciomarine sediments on the continental shelf off southeast Alaska and ice-rafted detritus in the abyssal North Pacific 3.5–3.0 Ma^45^ records late Pliocene expansion of glaciers in the St. Elias Mountains of Alaska, likely sometime between deposition of units 1 and 4. Expansion of glaciers adjacent to the Bering Sea is signalled by the first occurrence of sea-ice dinoflagellates at ~3.4 Ma^46^, shortly before or coincident with unit 1, and later by the first occurrence of sea-ice diatoms and common dropstones at ~2.7 Ma^46^. Increases in ice-rafted detritus derived from Greenland^11^ and East Antarctica^44^ during MIS KM2 and G22 indicate expansions of those ice sheets during the mid-Piacenzian warm period, coincident with deposition of units 3b, 3c, and 4. Repeated ice cap formation in the Cordillera Real from the end of the mid-Piacenzian warm period to the beginning of the Pleistocene (unit 4 and above; Figs 1 and 4) coincides with ice sheet expansion in Greenland from MIS G22 onward, the circum-North Atlantic from MIS G6 onward^11^, and Iceland possibly after ca. 3.0 Ma^41^. An incremental increase of ice sheet extent in East Antarctica appears to have commenced as early as MIS M2^47^. It is not clear whether the Cordillera Real experienced stepwise glacier expansion prior to the end of the mid-Piacenzian warm period. Glacial sediments older than ca. 3.05 Ma (units 1 to 3c) are exposed only at the Patapatani West section, and it is unclear how far south these glaciers extended. Both the Cordilleran Real record and oxygen isotope records lack the temporal resolution required to determine whether Pliocene glaciations in the South American tropics were truly synchronous with those at high northern latitudes, as reported for the Holocene^16^ or, alternatively, preceded Northern Hemisphere events, as apparently was the case during the Last Glacial Maximum^18^,^48^. Modeled orbital forcing suggests growth of the Antarctic Ice Sheet during the mid-Piacenzian while the Greenland Ice Sheet shrank^49^. Inter-hemispheric differences might also have allowed anti-phase growth of ice sheets in the Cordilleran Real relative to the Northern Hemisphere.

The formation and growth of ice caps in the Cordillera Real in the late Pliocene would have been accompanied by an expansion of treeless terrain in the currently forested parts of the eastern Central Andes, analogous to the savanna expansion during Pleistocene glaciations^50^. Similar altitudinal depression of forest biomes is well documented in the Northern Andes during the Early Pleistocene^43^. The formation of savanna-like environments in Central America and northern South America during Pleistocene glaciations has been recognized as an important factor in intercontinental faunal migrations of the Great American Biotic Interchange (GABI) starting at ca. 2.6 Ma^50^. Late Pliocene glaciation of the Cordillera Real may thus have played a previously unrecognized role in the exchange between North and South America of savanna-adapted mammals leading up to the GABI (Fig. 3h and Supplementary Information).

## Conclusions

The continuous glacial sequence at La Paz, Bolivia contains the only known record of low-latitude late Pliocene glaciation on Earth. Moreover, this sequence is probably the best terrestrial archive of Earth’s cryosphere during the mid-Piacenzian climatic optimum and subsequent end-Pliocene climatic deterioration. This record reveals that high-mountain ice caps formed repeatedly in the tropical Andes throughout the late Pliocene – before, during, and after the mid-Piacenzian warm period. The record pre-dates the onset of major, repeated tidewater glaciation (ca. 2.72 Ma, MIS G6) and widespread continental glaciation (ca. 2.64 Ma, MIS G2) that characterized the Northern Hemisphere during the Pleistocene. Direct evidence of an ice cap in the Central Andes during MIS M2 (ca. 3.3 Ma) adds important new data to the inventory of ice extent during this globally recognized cooling event. Good agreement with the best available high-latitude chronologies from the Northern and Southern Hemispheres indicates that late Pliocene glacial events in the southern tropics broadly coincided with those nearer both poles, as previously suggested^17^ for the Late Pleistocene. The Central Andean glacial record augments relatively limited Pliocene paleoenvironmental data from South America used in climate models and provides much-needed information on Pliocene terrestrial ice configurations. Notably, it implies late Pliocene environmental change in the Central Andes that may have contributed to the minor land mammal exchanges preceding the large-scale inter-American biotic exchange in the earliest Pleistocene. This unique glacial record by no means implies that the low-latitude cryosphere is unaffected by global warmth, such as that forecasted for the near future. Instead, it highlights the need for further work to elucidate the roles of paleotopography, ocean-climatic circulation, and moisture availability in the formation of tropical ice caps during Earth’s last global warm period.

## Methods

Laser ^40^Ar/^30^Ar step-heating was performed at the Oregon State University Argon Laboratory on sanidine and biotite recovered from a ~2-kg bulk sample of the Chijini Tuff at its type section on the east slope of the Río Kaluyo valley (16°25.87′ S, 68°8.04′ W, 4190 m asl) (Fig. 1c). We described and measured stratigraphy of road cuts on the west slope of the Río Kaluyo valley (16°25.37′ S, 68°08.09′ W; Fig. 1b) using field criteria, including texture, structure, lithology, colour, and weathering features. Unit boundaries were defined at contacts indicating depositional hiatuses, polarity reversals, or major changes in material and structural properties. Unit thickness and heights of key features above unit bases were measured with a handheld laser rangefinder. We measured bed thickness and dimensions of large clasts with a graduated metric scale. To characterize till fabrics, we measured the trend and plunge of 50 rod-shaped clasts (long-axis lengths greater than two times short-axis lengths) (Supplementary Figure S3). Six or more oriented, cylindrical samples (2.1 cm in diameter, 1.8 cm in length) were collected for paleomagnetic analysis from horizontally bedded lenses of silt or sand at each of 23 stratigraphic levels (Fig. 3b,c) throughout the sequence. Where such lenses were absent, we collected samples from the fine-grained matrix of diamicton or gravel, avoiding granules and pebbles. Chijini Tuff samples were taken from both the basal loose ash and overlying weakly cemented ash. All units identified during stratigraphic characterization, including all paleosols, were sampled. Each lithostratigraphic unit was sampled at several levels, except those less than 3 m in exposed thickness (units A and C2, Fig. 3a). We measured sampling heights above the section base with a handheld laser rangefinder. Samples were stored in magnetic shields following field collection and between measurements. We measured bulk magnetic susceptibility of each sample with a Sapphire Instruments SI-2B magnetic susceptibility and anisotropy meter. We measured natural remanent magnetization (NRM) of each of the 178 samples using an AGICO JR-6A spinner magnetometer. Remanence was re-measured after stepwise demagnetization in an alternating field (typically 5–20 steps ranging from 2.5 to 200 mT) using an ASC Scientific D-2000 alternating-field demagnetizer. We determined characteristic remanent magnetization (ChRM) directions by principal component analysis (PCA)^51^ using AGICO’s Remasoft v. 3.0 (paleomagnetic data analysis software). Mean remanence directions of sample groups were determined from PCA results of individual samples using the statistical module in Remasoft v. 3.0. We calculated mean directions for stratigraphic units (Fig. 4d; Supplementary Table S1) and overall means of normal and reversed polarity (Fig. 4c; Supplementary Table S2) in the same manner.

## Additional Information

**How to cite this article**: Roberts, N. J. *et al*. Multiple tropical Andean glaciations during a period of late Pliocene warmth. *Sci. Rep.* **7**, 41878; doi: 10.1038/srep41878 (2017).

**Publisher's note:** Springer Nature remains neutral with regard to jurisdictional claims in published maps and institutional affiliations.

## Supplementary Material

Supplementary Information
