# Supplementary material for: Multiple tropical Andean glaciations during a period of late Pliocene warmth
Source: Sci Rep. 2017 Feb 7;7:41878. doi: 10.1038/srep41878 (PMC5294413; doi:10.1038/srep41878)
Supplement: Supplementary Information [file srep41878-s1.pdf]

Multiple tropical Andean glaciations during a period of late Pliocene warmth (Supplementary Information)

Nicholas J. Roberts[1]*
René W. Barendregt[2]
John J. Clague[1]

[1]Department of Earth Sciences, Simon Fraser University, 8888 University Drive, Burnaby, Canada, V5A 1S6
[2]Departmetn of Geography, University of Lethbridge, 4401 University Drive West, Lethbridge, Canada, T1K 6T5
*Correspondence to nickr@sfu.ca

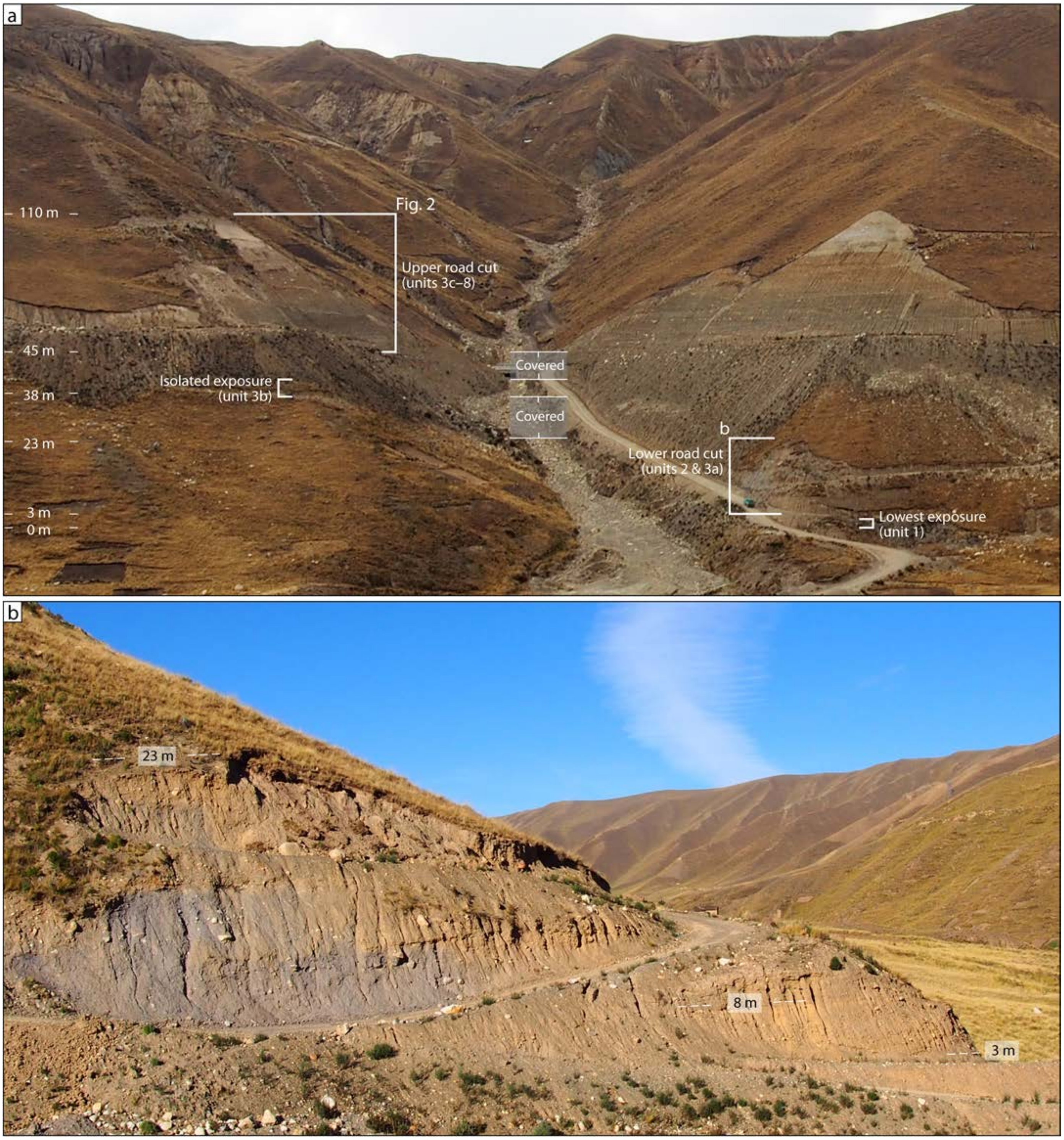

Supplementary Figure S1. Overview of the Patapatani West section. a. Patapatani West section viewed from the Patapatani East section across the Río Kaluyo valley. Heights at the left side of the photo indicate the boundaries of parts of the section above the base of unit 1. **b.** The lower road cut at the Patapatani West section, exposing 20 m of massive to weakly stratified diamicton overlying 2 m of poorly sorted gravel. The exposure shown here spans the zone from 3 m to 21 m in Figure 3, comprising most of unit 2 (between 3 and 8 m height) and unit 3a (between 8 and 23 m height); the lowest 3 m of the section, including all of unit 1, are not visible. See Figure 2 for an overview of the upper part of the stratigraphic sequence.

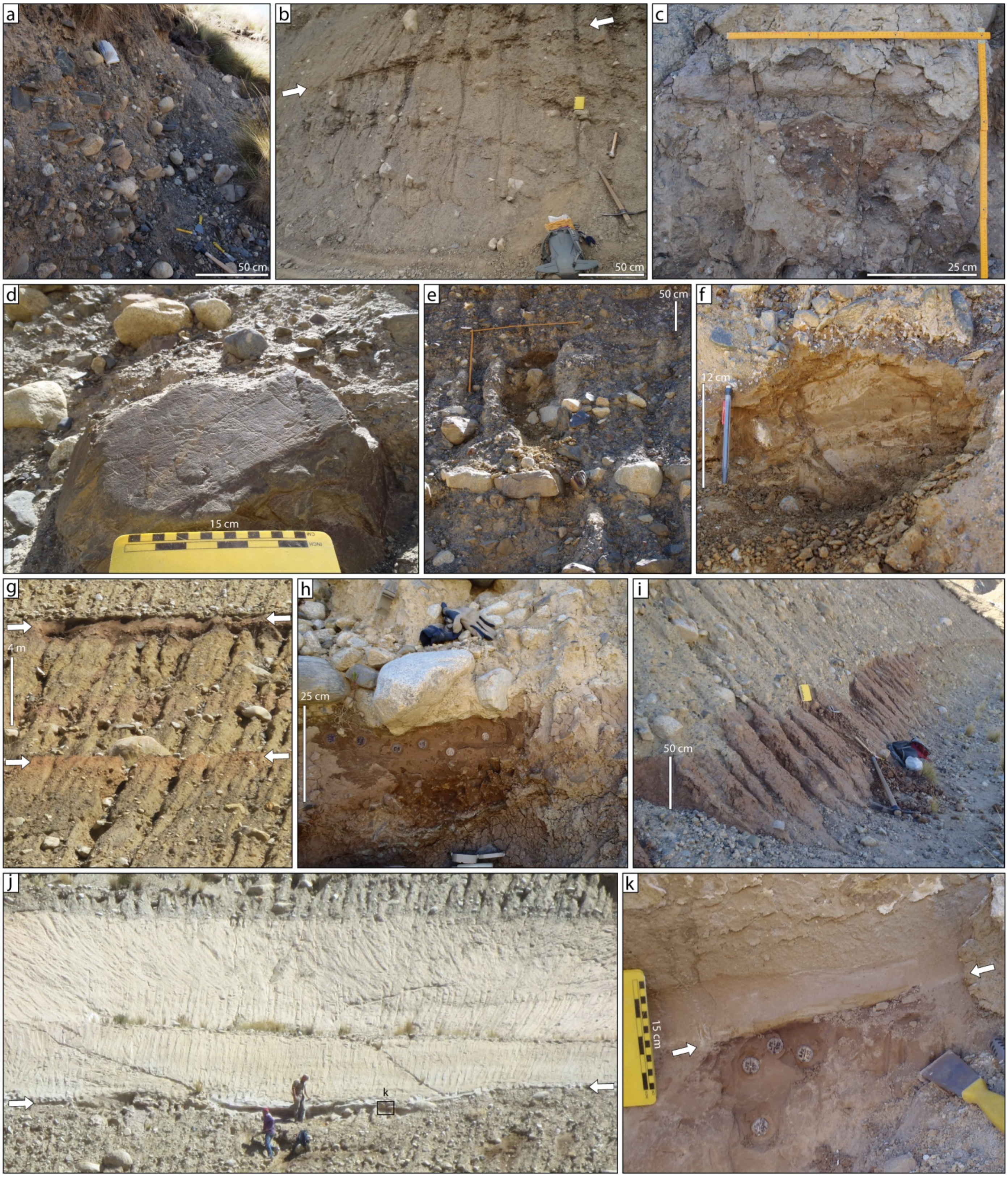

a
50 cm
b
50 cm
c
25 cm
d
15 cm
e
50 cm
f
12 cm
g
4 m
h
25 cm
i
50 cm
j
k
k
15 cm

Supplementary Figure S2. Examples of sediments at the Patapatani West section. a. Poorly sorted, weakly stratified gravel at the base of the section (unit 1). **b.** Gently dipping, stratified, matrix-supported diamicton (unit 2) and overlying matrix-supported diamicton (unit 3a) separated by an angular unconformity (paired white arrows)**. c.** Matrix-supported diamicton (unit 3a). **d.** Glacially striated phyllite clast within diamicton (unit 3c). **e.** Weakly stratified, matrix-supported diamicton with faceted granite and phyllite clasts (unit 4). **f.** Laminated silt lens within weakly stratified diamicton (unit 4). **g.** Three weakly stratified diamictons (units 4-6) with cobble-boulder stringers and separated by paleososl (paired white arrows). **h.** Contact between two weakly stratified diamictons (units 5 and 6) with a well developed paleosol (top of unit 5) overlain by a cobble stringer (base of unit 6). **i.** Contact between two weakly stratified diamictons (units 6 and 7) with a well developed, laterally extensive paleosol. **j.** Laterally extensive tuff (unit 8) comprising a thin zone of loose friable ash (shadowed, recessive zone between white arrows) overlain by weakly cemented, cliff-forming ash. Note people (1.8 m tall) for scale. **k.** Lower part of tuff (unit 8) showing loose friable ash, overlying weakly cemented ash, and base of transition (paired white arrows) between them. See box in panel j for location.

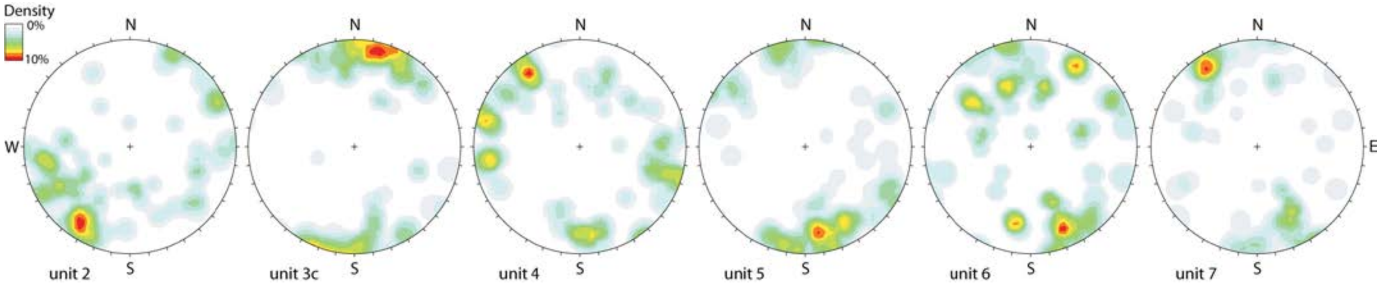

Supplementary Figure S3. Diamicton clast fabrics for select glacial units. Suggested ice-flow directions are to the south-southwest (units 2 and 3c) and south-southeast (units 4, 5, 6, and 7). The fabrics are based on trends and plunges of 50 elongate stones (long:short axis ratio of ≥2:1) and are represented by Fisher distributions on equal-angle stereonets.

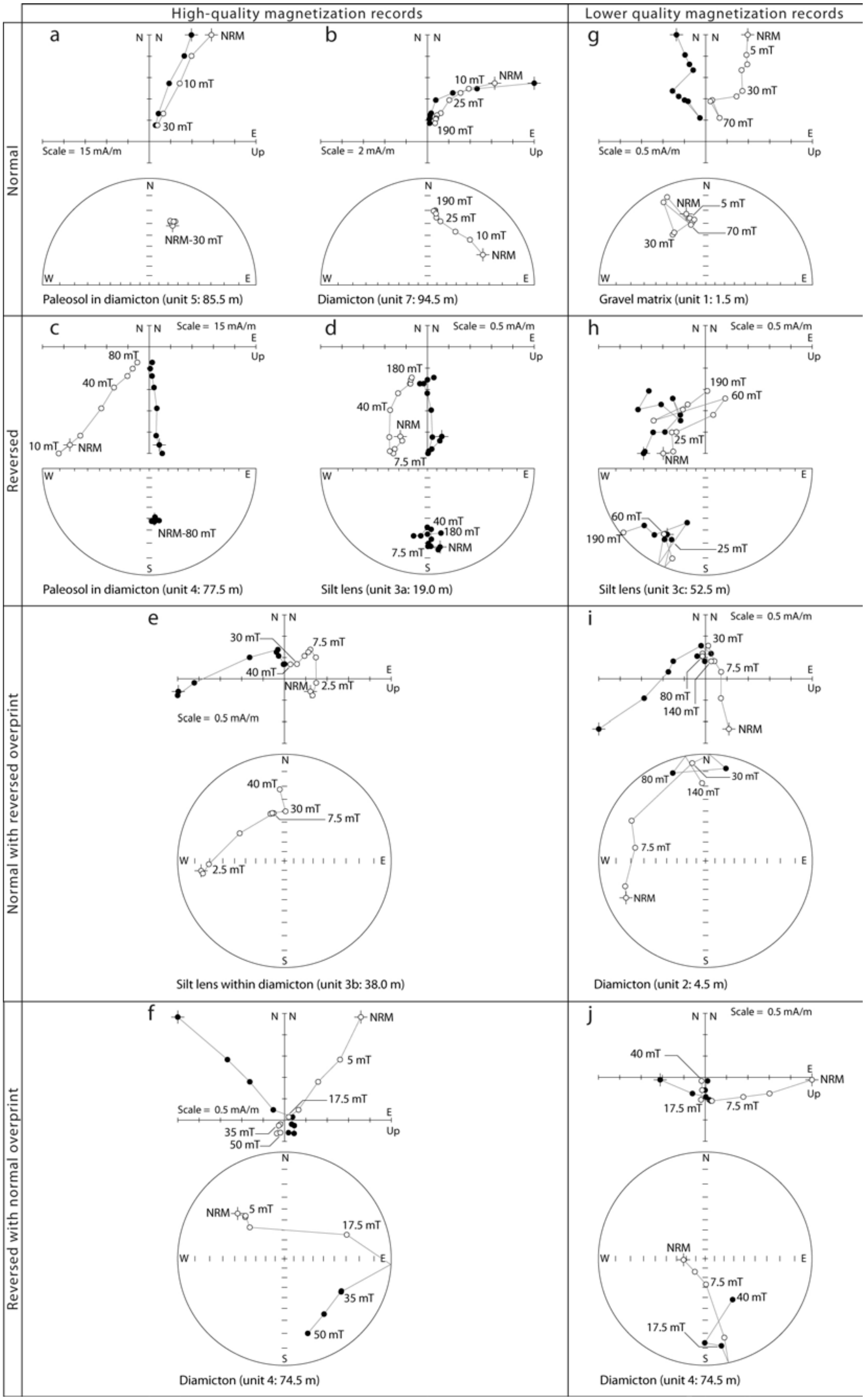

High-quality magnetization records
Lower quality magnetization records
Normal
Reversed
Normal with reversed overprint
Reversed with normal overprint
a
Scale = 15 mA/m
Paleosol in diamicton (unit 5: 85.5 m)
NRM-30 mT
b
Scale = 2 mA/m
Diamicton (unit 7: 94.5 m)
g
Scale = 0.5 mA/m
Gravel matrix (unit 1: 1.5 m)
c
Scale = 15 mA/m
NRM-80 mT
Paleosol in diamicton (unit 4: 77.5 m)
d
Scale = 0.5 mA/m
Silt lens (unit 3a: 19.0 m)
h
Scale = 0.5 mA/m
Silt lens (unit 3c: 52.5 m)
e
Scale = 0.5 mA/m
Silt lens within diamicton (unit 3b: 38.0 m)
i
Scale = 0.5 mA/m
Diamicton (unit 2: 4.5 m)
f
Scale = 0.5 mA/m
Diamicton (unit 4: 74.5 m)
j
Scale = 0.5 mA/m
Diamicton (unit 4: 74.5 m)

Supplementary Figure S4. Sample behaviour during stepwise AF demagnetization. Solid and open symbols on orthogonal plots indicate, respectively, horizontal and vertical planes; solid and open symbols on corresponding stereo plots indicate lower-hemisphere and upper-hemisphere projections. Natural remanent magnetization (NRM) is shown with the cross-haired circle. Representative samples are shown for normal magnetization (a, b, and g); reversed magnetization (c, d, and h); and normal magnetization with a reversed overprint (e and i); and reversed magnetization with normal overprint (e and j).

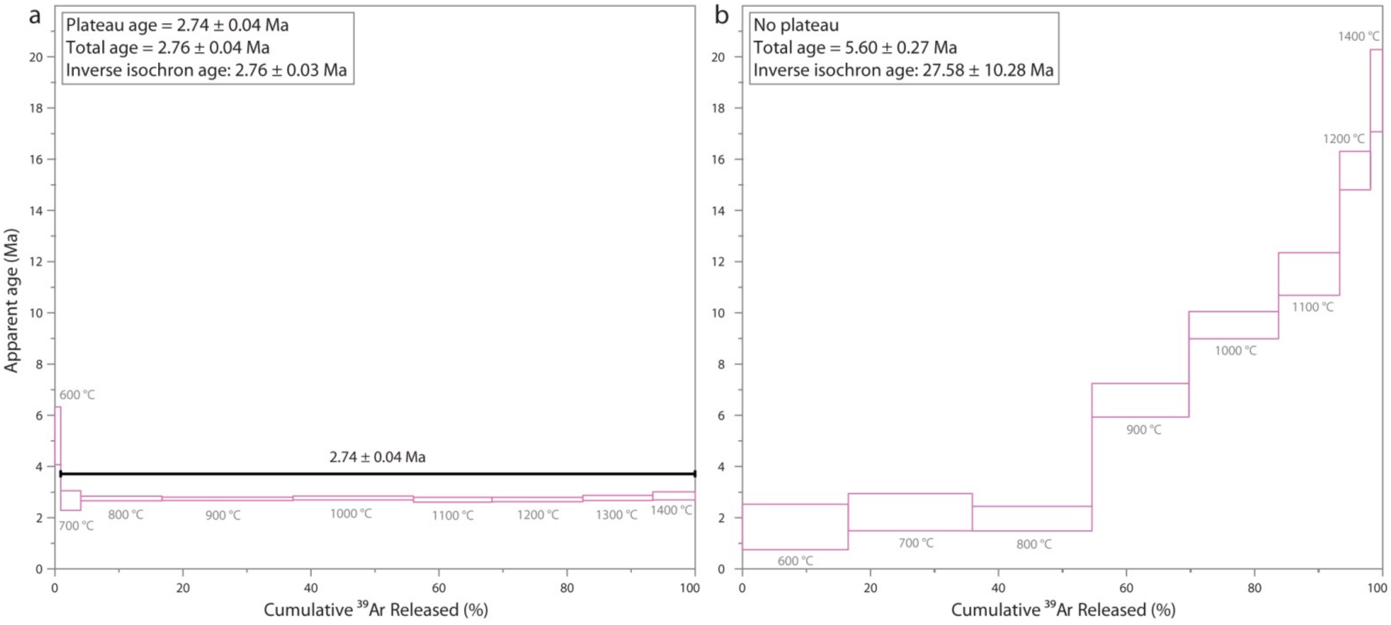

Supplementary Figure S5. $^{40}Ar/^{39}Ar$ age spectra for the dated sample of Chijini Tuff (16° 25.87' S, 68° 8.04' W, 4190 m asl). **a.** Sanidine. **b.** Biotite.

Supplementary Table S1. Paleomagnetic directions by stratigraphic unit.

Unit	Lithology	χ	n			*D*	*I*	*k*	α_{95}	p
Level (m) [a]	Material		Collected	Useful [b]	Used [c]					
**Unit 8**	**Chinjini Tuff**									
101.0	Weakly cemented silty ash	573	6	6	6	10.8	-37.2	90	7.1	N
100.0	Weakly cemented silty ash	686	6	6	3	0.9	-27.1	364	6.5	N
97.0	Weakly cemented silty ash	729	6	6	6	15.6	-28.7	69	8.1	N
95.5	Loose silty ash	407	6	6	5	6.7	-44.1	70	9.1	N
			**24**	**24**	**20**	**11.5**	**-34.9**	**57**	**3.5**	**N**
**Unit 7**	**Patapatani Formation**									
94.5	Diamicton	119	6	6	6	0.5	-13.2	37	11.2	N
92.0	Diamicton	74	6	6	6	8.3	-29.6	28	12.9	N
91.0	Diamicton	284	6	6	6	355.9	-36.9	66	8.3	N
			**18**	**18**	**18**	**1.7**	**-26.7**	**24**	**7.2**	**N**
**Unit 6**	**Patapatani Formation**									
89.5	Paleosol formed in diamict	2368 *	6	6	6	6.0	-43.6	251	4.2	N
88.0	Diamicton	85	6	6	5	28.4	-27.4	21	17.2	N
			**12**	**12**	**11**	**17.1**	**-36.9**	**22**	**9.9**	**N**
**Unit 5**	**Patapatani Formation**									
85.5	Paleosol formed in diamict	5556 *	6	6	6	19.8	-28.0	344	3.6	N
81.5	Diamicton	155	6	4	3	347.7	-23.2	67	15.2	N
			**12**	**10**	**9**	**9.0**	**-27.2**	**27**	**10.2**	**N**
**Unit 4**	**Patapatani Formation**									
77.5	Paleosol formed in diamict	2641 *	6	6	4	182.4	42.5	82	10.2	R
74.5	Diamicton	125	6	6	5	166.2	14.6	143	6.4	R
70.0	Sand lens	168	6	4	3	217.0	55.6	42	19.4	R
62.0	Silt lens	162	6	6	4	182.7	1.6	31	16.7	R
			**24**	**22**	**16**	**180.7**	**26.8**	**9**	**12.9**	**R**
**Unit 3c**	**Patapatani Formation**									
57.5	Paleosol (weakly developed)	154	6	4	4	213.3	70.0	8	34.8	R
52.5	Silt lens	100	14	9	6	187.6	28.8	20	15.4	R
			**20**	**13**	**10**	**192.6**	**44.9**	**7**	**19.5**	**R**
**Unit 3b**	**Patapatani Formation**									
38.0	Silt lens	149	8	8	6	356.3	-28.4	56	9.1	N
**Unit 3a**	**Patapatani Formation**									
19.0	Silt lens	127	12	9	9	191.1	26.0	8	19.7	R
9.5	Sand lens & diamict	146	16	12	10	164.9	67.5	31	8.9	R
			**28**	**21**	**19**	**182.2**	**49.6**	**7**	**13.8**	**R**
**Unit 2**	**Pre-Patapatani stratified diamict**									
7.0	Sand lens	293	6	6	6	358.2	-32.4	173	5.1	N
4.5	Silt lens & diamict	158	12	11	8	5.4	-15.7	31	10.1	N
			**18**	**17**	**14**	**2.5**	**-23.0**	**31**	**7.3**	**N**
**Unit 1**	**Pre-Patapatani gravels**									
1.5	Gravel matrix	113	6	5	4	359.1	-15.2	15	24.6	N
		**Total**	170	150	127					

Notes: χ, mean magnetic susceptibility (10-6 SI units/vol.) of collected samples; N, number of samples; D and I, mean declination and inclination; k, precision parameter; α_{95}, circle of confidence (P = 0.05); p, polarity.

* Enhancement of ferromagnetic content within paleosol.

[a] Sampling height in metres above base of section.

[b] Polarity determination.

[c] Included in calculation of directional means.

Supplementary Table S2. Mean paleomagnetic directions.

Grouping	n	*D*	*I*	*k*	*α95*
Normal-polarity samples	82	5.9	-29.2	24.74	3.2
Reverse-polarity samples	45	183.7	40.4	7.06	8.6
All specimens*	127	5.2	-32.9	12.59	3.7
Normal-polarity units	7	4.8	-27.6	71.41	7.2
Reverse-polarity units	3	184.9	40.6	39.06	20
All units*	10	4.8	-31.5	9.81	7.1

Notes: n, number of samples/units; D and I, mean declination and inclination; k, precision parameter; α_{95}, circle of confidence (P = 0.05). Mean inclination expected for a Geocentric Axial Dipole (GAD) at this sampling latitude: -30.5°/30.5°. Earth's present magnetic field (PEF) direction at sampling locality: D = 352.5°, I = -10.1.

* Irrespective of sign (upper hemisphere).

Supplementary Table S3. Average aggradation rates of Plio-Pleistocene continental fill sequences in the Central Andes.

Sequence	Thickness (m)	Age (ka) Base	Top	Range	Aggradation rate (cm/ka)	Limiting ages*	Geologic age	Source
**La Paz basin**								
Patapatani West (units 3-7)	87	3319	2740	579	15.0	Start of Mammoth to 2.74 Ma	Zanclean-Piacenzian	This study
Patapatani West (units 1-7)	95	3340	2740	600	15.8	MIS MG2 to 2.74 Ma	" "	" "
Patapatani West (units 1-7)	95	3380	2740	640	14.8	MIS MG4 to 2.74 Ma	" "	" "
Viscachani	135	3588	2740	848	15.9	End of Gilbert to 2.74 Ma	Zanclean-Piacenzian	Thouveny & Servant (1989)
**Elsewhere**								
Inchasi section, Bolivia	80	4184	3319	865	9.2	End of Cochiti to start of Mammoth	Zanclean-Piacenzian	MacFadden et al. (1993)
Uquía Formation, Argentina	140	2608	1781	827	16.9	Gauss-Matuyama boundary to end of Olduvai	Gelasian	Marshall et al. (1982)
Tarija basin, Bolivia	50	1075	780	295	16.9	Start of Jaramillo to Matuyama-Brunhes boundary	late Calabrian	MacFadden et al. (1983)

* With the exception of Marine Isotope Stages (MIS) and our revised age for the Chijini Tuff, limiting ages of the fill sequences are based on astronomically tuned Chron and subchron boundaries[13] (Figure 5c).

Paleosol characteristics and duration of formation

We interpret the four reddened zones (e.g. Supplementary Figure S2g-i) in the upper part of the Patapatani West section to be paleosols. Each is laterally extensive over the 40-100 m width of the outcrop, and each has an abrupt planar upper contact. The reddish colour gradually transitions downward over 0.1-0.5 m into the typical unaltered olive-gray colour of the diamicton. The clast content of the diamicton increases downward through the reddish zones. Each zone has a vertical columnar or prismatic structure, and clay skins are present on column or block surfaces and on some clasts.

The zones also have higher magnetic susceptibility values than the bounding olive-gray diamicton (Figure 3b; Supplementary Table S1), reflecting elevated ferrimagnetic mineral content[52], particularly magnetite[53]. Magnetic enrichment is common in interglacial soils (p. 46 in reference 53), for example in the well drained, non-acidic paleosols within loess sequences in China, Central Asia, and Europe[54]. Magnetic susceptibility maxima in those loessic paleosols[54,55] and in paleosols in general[56,57] are due largely to authigenic production and enrichment of ferrimagnetic minerals during pedogenesis (pp. 69-73 in reference 58), including production by magnetic bacteria[59,60].

The physical and magnetic characteristics described above are associated with both surface and buried soils[57,61,62]. We thus infer that the reddened zones are paleosols and record lengthy periods of subaerial exposure and weathering, during which the ground surface was stable. Silicate clay accumulation, rubification, and ped development reflect formation of an argillic (Bt) horizon, indicative of mature soils[62]. Prismatic Bt-horizon peds like those we observed (Supplementary Figure S2g-i) specifically suggest formation in arid to semi-arid climates (p. 135 in reference 62).

Because climate, vegetation, and parent material strongly influence pedogenesis, the length of time over which these paleosols developed can be only roughly approximated by comparison with other soils of similar type and maturity. Under sub-humid to humid conditions Bt horizons are suggested to form over periods of several thousand to several tens of thousands of years[62]. The buried soils capping Patapatani till units may record periods nearer the upper end this range, given that they formed at high elevations and perhaps in a relatively dry environment.

In the Rocky Mountains, northern Montana, well developed Bt horizons in glacial and glaciofluvial parent materials appear to have formed over periods much longer than the Holocene[63]. Soils that have formed in this setting since the Last Glacial Maximum, about 20,000 years ago, typically lack argillic horizons, and those with clay enrichment have thin (15-25 cm; mean 19 cm) weak Bt horizons. In contrast, soils formed since MIS 6 or possibly MIS 4 have thicker (34-92 cm; mean 58 cm) Bt horizons, similar those described here.

Soil formation of the degree observed at the tops of units 3, 4, 5 and 6 requires non-glacial conditions providing stable sub-aerial landscapes and mild climatic over substantial periods. The brief intervals between advances of the same glaciation – particularly the very short warm intervals of cold peaks during 40-ka late Pliocene climate cycles – would be insufficient in length and likely degree of warming, to develop prominent Bt horizons. We thus suggest that each of the paleosols of the Patapatani sequence records passage of an interglacial period on the order of 10^3, but most likely if not 10^4 years.

Paleomagnetic results

Sampled sediments at the Patapatani West section are, for the most part, strongly magnetic (magnetic susceptibility values range from 50 to 6000 x 10^{-6} SI units/vol.), probably due to magnetic minerals sourced from plutons in the Cordillera Real and volcanic beds of the fill sequence underlying the Altiplano. The ranges of magnetic susceptibility values in glacial and volcanic deposits are, respectively, 75-300 x 10^{-6} and 450-600 x 10^{-6} SI units. The magnetic susceptibilities of paleosols are higher than those of the tills in which they have formed and are especially high (2000-6000 x 10^{-6} SI units) in the best-developed paleosols (upper contacts of units 4, 5, and 6; Supplementary Table S1).

The magnetic stability of the samples is relatively high, with 71% (127 of 178) of all samples and 70% (103 of 148) of samples from diamicton units providing stable, coherent remanent magnetization directions. In other paleomagnetic studies of glacial diamictons, up to 50% of samples are commonly rejected because of weak or unstable magnetizations or because of incoherent remanence directions held by randomly oriented sand grains or pebbles within the sample[38]. Of the 127 samples used in our study, about half provided high-quality magnetization records (Supplementary Figure S4a-f) that provided precise determination of a characteristic (primary) remanent magnetization (ChRM) direction. The remainder provided records of moderate quality (e.g. Supplementary Figure S4g-j) that gave less precise, but still useful, ChRM directions.

The 51 samples (29% of the sample collection) that we did not use in the calculation of directional means were not restricted to any particular unit. These samples either revealed incoherent directions during AF demagnetization, which prevented confident polarity assignment, or had high errors (maximum angular deviation [MAD] of 6 to 20°). Half (25) of the unused samples also exhibited a very low natural remanent magnetization (< 0.5 mA/m), which may explain their irregular demagnetization behaviour.

Most samples reveal magnetizations typical of magnetite, with median destructive fields (MDF) ranging widely from 10 to 80 mT. The majority of these samples appear to contain mainly single-domain magnetite (MDF between 20 and 80 mT); about one-third exhibit soft magnetization indicative of multi-domain magnetite (MDF <20 mT). Several samples have multi-component magnetizations, reflecting the presence of both single-domain magnetite and hematite grains. A few samples (from the top of unit 7) have MDF values higher than the maximum AF demagnetization step (200mT) used in this study, suggesting that they contain predominantly hematite.

Alternating-field demagnetization removed viscous components of magnetization, revealing single-component directions that reflect paleo-field directions (Figure 4a-b; Supplementary Figure S4). The sample collection includes both normal and reversed polarities (Supplementary Tables S1 and S2; Figure 4b). The mean direction of all normally magnetized samples is within 1.5° of the Geocentric Axial Dipole (GAD) field at the sampling latitude whereas the mean direction of all reversely magnetized samples is 11° steeper than the GAD (Supplementary Table S2; Figure 4c) and may reflect incomplete removal of overprints at the maximum cleaning field. A few samples exhibit low coercivity overprints (removed between 7.5 and 30 mT AF demagnetization), acquired subsequently in a field of opposite polarity (Supplementary Figure S4e, f, I, and j).

All units can be characterized as being either normally or reversely magnetized, with mean directions clustered near a normal or reversed GAD position for the sampling latitude (Figure 4d). All magnetozones identified in the Patapatani West section are represented by samples collected at multiple levels (Figure 3c), except for the one corresponding to unit 3b (magnetozone N2), which is based on a single sample group (Figure 3c at 38 m) taken across the gulley from the main exposure. The stratigraphic position of unit 3b is well constrained because the

sequence is spatially extensive and nearly horizontal. Unit 3b is likely in situ because it records coherent paleomagnetic directions (e.g. Supplementary Figure S4e) that align with the GAD direction for a normal field at this latitude (Figure 4d), as well as with other normally magnetized units in this study (Supplementary Table S1; Figure 4d). Additionally, archival imagery of the slope shows no evidence of mass movement. The presence of a short normally magnetized interval of similar stratigraphic position at the Viscachani section 6.5 km south (Figure 5) further supports the in situ nature of unit 3b.

Biotite alteration and radiometric dating of Altiplano tuffs

Clapperton[64] suggested that the large difference in radiometric ages from tuffs in the northern part of the La Paz basin – 3.27 ± 0.14 Ma and 3.28 ± 0.13 Ma[22] compared with 2.650 ± 0.012 Ma[24] to 2.8 ± 0.1 Ma[23] – is due to the presence of a second, pre-Chijini tuff preserved nowhere else in the area. However, multiple lines of evidence instead suggest that the initial age estimate for the Chijini Tuff of 3.28-3.27 Ma[22] is erroneous due to biotite alteration. The true age of the Chijini Tuff is narrowly constrained by multiple reliable K-Ar and $^{40}Ar/^{39}Ar$ analyses to ca. 2.8-2.65 Ma.

Biotite recovered from tuffs in the Altiplano region typically yields older ages than feldspar[23, 24]. Previous K-Ar dating of the Chijini Tuff from the west slope of the Río Choqueyapu valley, 5 km downstream of the section described here (Figure 1), yielded an age of 2.8 ± 0.1 Ma for potassium feldspar, but ages of 12.1 ± 1.1 and 11.6 ± 1.3 Ma for biotite[23]. Single-crystal total fusion $^{40}Ar/^{39}Ar$ analysis of a sample of Chijini Tuff from the Río Chuquiaguillo valley, 5 km southeast of our section (Figure 1), yielded average ages for sanidine and biotite of 2.650 ± 0.012 Ma and 3.082 ± 0.029 Ma, respectively[24].

Single-crystal laser step-heating of tuffs from Wyoming Basin[65] characterizes the influence of biotite alteration on radiometric age determination. In their study, unaltered biotite yielded concordant $^{40}Ar/^{39}Ar$ plateau ages indistinguishable from those yielded by sanidine. Altered (potassium-depleted) biotite, however, yielded discordant age spectra with age plateaus 1-14% older that those yielded by sanidine. The integrated (total-fusion) ages of the altered biotite show increasing scatter with spectra discordance. Thus, altered biotite crystals are unsuitable for high-resolution dating applications[65].

Single-crystal laser step-heating of the Chijini Tuff sample we collected from the Río Kaluyo valley also showed a sanidine-biotite age disparity. Sanidine yielded a concordant $^{40}Ar/^{39}Ar$ age spectrum with a weighted age plateau at 2.74 ± 0.04 Ma (2σ uncertainty) (Supplementary Figure S5a). Biotite, which was altered (Dan Miggins, Oregon State University, personal communication, 2013), produced a discordant age spectrum; age steps increased with temperature from 1.65 ± 0.89 Ma (at 600 °C) to 18.65 ± 1.61 Ma (at 1400 °C) and did not plateau (Supplementary Figure S5b). Integrated ages for our sanidine (2.76 ± 0.04 Ma [2σ uncertainty]) and biotite (5.60 ± 0.27 Ma [2σ uncertainty]) fractions show that the latter overestimate the age of the Chijini Tuff by an amount similar to that previously noted by pervious dating studies[23,24].

Plio-Pleistocene sediment aggradation rates in the Central Andes

We estimate long-term average aggradation rates based on the magnetostratigraphic data from the Patapatani West section (Supplementary Table S3). There are 88 m of diamicton between the lowest polarity reversal at the Patapatani West section (8 m above the base of the section) and the base of the Chijini Tuff. The average aggradation rate for units 3a to 7 thus is 15 cm/ka. If unit 1 records MIS MG4 (ca. 3.380 Ma[13]), the average

aggradation rate of the full 95 m sub-Chijini sequence is likewise 15 cm/ka. Alternatively, if unit 1 was deposited during MIS MG2 (ca. 3.340 Ma[13]), the average aggradation rate of the sub-tuff section is closer to 16 cm/ka.

The sequence records very rapid punctuated accumulation of diamictons during glaciations separated by long periods of landscape stability during interglacials. The average rates thus provide little insight on rates of landscape modification on the scale of glacial-interglacial cycles. They do, however, facilitate comparison of long-term in-fill within and between tectonic basins. A nearly identical average aggradation rate is suggested for Thouveny and Servant's[21] Viscachani section (Supplementary Table S3) based on the polarity reversal at the Gilbert-Gauss boundary (3.588 Ma) and the age presented here for the Chijini Tuff. This close agreement adds credence to our proposed chronology for the Patapatani West section and suggests that sedimentation several kilometres beyond the ice margin kept pace with glacially dominated sedimentation nearer the Cordillera Real.

Late Pliocene aggradation in the La Paz basin is within the range of long-term aggradation rates recorded by Pliocene and Pleistocene sequences elsewhere in the Central Andes (Supplementary Table S3). Early Pleistocene sections along the Andean front at Uquía, Argentina[66], and Tarija, Bolivia[67], indicate average aggradation rates (17 cm/ka) nearly identical to those at the Patapatani West and Viscachani sections. The Inchasi section near Potosí, Bolivia[68], spans the middle part of the Pliocene, including the period recorded by units 1 and 2 and possibly unit 3a of the Patapatani West section. Its long-term average aggradation rate is 9 cm/ka. Stage-scale basin aggradation across the eastern Central Andes appears to have been roughly similar during the Plio-Pleistocene, possibly suggesting an overall tectonic control on basin infill. In contrast, relatively rapid (~90 cm/ka) aggradation of the middle Miocene Corque basin in the central Altiplano (~100 km south-southwest of La Paz) suggests an increased tectonic role leading to active subsidence from ca. 12 to 9 Ma[69].

Faunal interchange during early Central Andean glaciation

Isolated faunal evolution in South America during the Paleogene produced unique land mammal groups[70]. In the Miocene, an exchange of North and South American taxa began with the gradual closing of the Central American Seaway, which was complete by ca. 2.8 Ma[71]. These exchanges culminated in the Great American Biotic Interchange (GABI)[72], comprising four main phases of inter-continental migration between ca. 2.8 and 0.125 Ma [50,73,74].

Although the formation of the Isthmus of Panama[71] is required to facilitate large-scale inter-American exchange of land mammals, it does not alone explain the GABI[50,74,75]. The dense vegetation of the region's modern biomes forms a barrier to rapid, long-distance migration[50]. By contrast, open environments necessary for large-scale faunal exchange between the Americas occurred during glaciations due to the formation of savanna-like corridors that included land exposed by eustatic sea-level depression[50,75,76]. These corridors may have been particularly well developed when the Laurentide Ice Sheet developed in the earliest Pleistocene[75]. End-Pliocene global cooling thus set the stage for the first major pulse of the GABI at ca. 2.6-2.4 Ma[50,73,74], which occurred shortly after the initiation of major Northern Hemisphere glaciation (2.75 Ma[12]).

Land mammal exchanges leading up to the GABI may in part reflect the formation of similar corridors in the late Pliocene. These earlier corridors would have been more limited due to lesser establishment of the Isthmus of Panama[50,71] and weaker global glacial cycles[13] accompanied by smaller eustatic sea-level drops and lesser equatorial biome changes. Open savanna-like environments in the Central Andes may have coexisted with local ice caps, aiding these pre-GABI migrations.

Several savanna-adapted North American families reached South America during the intensification of glacial cycles in the Cordillera Real (Figure 3h), although in many cases limited geochrologic control prevents precise dating of their arrival. Camelids (family Camelidae) spread to South America ca. 3.3 Ma[73,77]. Savanna-adapted peccaries reached South American no later than 3.1 Ma[50,78] – *Platygonus* first appears in the Pampas region south of the Central Andes around the start of the late Pliocene[78,79], and *Catagonus* appears before the end of the Pliocene[79]. Rodents of the family Cricetidae and carnivorous mammals of the family Mustelidae arrived, respectively, in the early and late parts of the late Pliocene[77]. Equids arrived sometime in the late Pliocene as well[78].

Two major endemic South American genera migrated north during this same late-Pliocene time window (Figure 3h). *Glossotherium* (a ground sloth) reached Arizona and Florida by ca. 3.0 Ma[50,80]. *Glyptotherium* (a glyptodont) arrived in Mexico in the Pliocene, most likely during the latest Gauss[81]; it spread farther north by ca. 2.7 Ma[50], roughly coincident with the first cold peak (MIS G6) following the glacial record reported here.

Additional references

52. Butler, R.F. *Paleomagnetism: Magnetic Domains to Geologic Terrains*, 319 pp. (Blackwell Scientific Publications, London, 1992).

53. Opdyke, N.O. & Channell, J.E.T.. *Magnetic Stratigraphy*. Intern. Geophys. Ser. 64, 346 pp. (Academic Press, London, 1996).

54. Maher, B. Magnetic properties of modern soils and Quaternary loessic paleosols: Paleoclimatic implications. *Palaeogeogr. Palaeoclimatol. Palaeoecol.* **137**, 25-54 (1998).

55. Eyre, J.K. & Shaw, J. Magnetic enhancement of Chinese loess-the role of $_{\gamma}Fe_2O_3$? *Geophys. J. Int.* **117**, 265-271 (1994).

56. Mullins, C.E. Magnetic susceptibility of the soil and its significance in soil science - A review. *J. Soil Sci.* **28**, 223-246 (1977).

57. Catt, J.A. Field recognition, description and spatial relationships of paleosols. *Quat. Int.* **6**, 2-95 (1990).

58. Evans, M.E. & Heller, F. Environmental Magnetism: Principles and Applications of Enviromagnetics. 299 pp. (Academic Press, San Diego, 2003).

59. Fassbinder, J.W.E., Stanjek, H. & Vali, H. Occurrence of magnetic bacteria in soil. *Nature* **343**, 161-163 (1990).

60. Evans, M.E. & Heller, F. Magnetic enhancement and palaeoclimate: study of a loess/palaeosol couplet across the Loess Plateau of China. *Geophys. J. Int.* **117**, 257-264 (1994).

61. Retallack, G.J. *Field Recognition of Paleosols*. Geol. Soc. Am. Spec. Pap. **216**, 1-20 (1988).

62. Brady, N.C. & Weil, R.R. *The Nature and Properties of Soil*, 32th ed. 960 pp. (Prentice Hall, Upper Saddle River, NJ, 2002).

63. Karlstrom, E.T. Use of soils to identify glacial deposits of various ages east of Glacier National Park, Montana, U.S.A. *Arct., Antarc., Alp. Res.* **32**, 179-188 (2000).

64. Clapperton, C.M. Late Cenozoic glacial history of the Andes, Part I: Late Miocene-Middle Pleistocene. In *Quaternary Geology and Geomorphology of South America* (ed. Clapperton, C.M.). 335-354 (Elsevier Science, Amsterdam, 1993).

65. Smith, E.M., Singer, B.S., Carroll, A.R. & Fournelle, J.H. Precise dating of biotite in distal volcanic ash: Isolating subtle alteration using $^{40}Ar/^{39}Ar$ laser incremental heating and electron microprobe techniques. *Am. Mineral.* **93**, 784-795 (2008).

66. Marshall, L.G., Butler, R.F., Drake, R.E. & Curtis, G.H., 1982. Geochronology of type Uquian (Late Cenozoic) land mammal age, Argentina. *Science* **216**, 986–989 (1982).

67. MacFadden, B.J., Siles, O., Zeitler, P., Johnson, N.M. & Campbell, K.E. Magnetic polarity stratigraphy of the middle Pleistocene (Ensenadan) Tarija formation of southern Bolivia. *Quat. Res.* **19**, 172-187 (1983).

68. MacFadden, B.J., Anaya, F. & Argollo, J. Magnetic polarity stratigraphy of Inchasi – A Pliocene mammal-bearing locality from the Bolivian Andes deposited just before the Great American Interchange. *Earth Planet. Sci. Lett.* **114**, 229-241 (1993).

69. Roperch, P., Hérail, G. & Fornari, M. Magnetostratigraphy of the Miocene Corque basin, Bolivia: Implications for the geodynamic evolution of the Altiplano during the late Tertiary. J. Geophys. Res. **104 (B9)**, 20,415-20,429 (1999).

70. Simpson, G.G. *Splendid isolation: the curious history of South American mammals.* 266 pp. (Yale University Press, New Haven, 1980).

71. O'Dea, A. *et al*. Formation of the Isthmus of Panama. *Sci. Adv.* **2**, e1600883 (2016).

72. Marshall, L.G., Bulter, R.F., Drake, R.E. Curtis, G.H. & Tedford, R.H. Calibration of the Great American Interchange. *Science* **204,** 272-279 (1979).

73. Cione, A.L. *et al. Mamíferos continentales del Mioceno tardío a la actualidad en la Argentina: cincuenta años de estudios*. (Publicación Especial 11, Asociación Paleontológica Argentina. Ameghiniana 50° aniversario, Buenos Aires, 22 pp, 2007).

74. Cione, A.L., Gasparini, G.M., Soibelzon, E., Soibelzon, L.H. & Tonni, E.P. *The Great American Biotic Interchange: A South American Perspective.* 97 pp. (Springer, 2015).

75. Bacon, C.D. *et al*. Quaternary glaciation and the Great American Biotic Interchange. *Geology* **44**, 375-378 (2016).

76. Webb, S.D. Ecogeography and the Great American Interchange. *Paleobiology* **17,** 266-280 (1991).

77. Cione, A.L. & Tonni, E.P. Chronostratigraphy and "Land-Mammal Ages" in the Cenozoic of southern South America: Principles, practices, and the "Uquian" problem. *J. Paleontol.* **69,** 135-159 (1995).

78. Prevosti, F.J., Gasparini, G.M. & Bond, M. On the systematic position of a specimen previously assigned to Carnivora from the Pliocene of Argentina and its implications for the Great American Biotic Interchange. *Neues Jahrbuch. Geol. P.-A.* **242,** 133-144 (2006).

79. Gasparini, G.M. Records and stratigraphical ranges of South American Tayassuidae (Mammalia, Artiodactyla). *J. Mammal Evol.* **20,** 57-68 (2013).

80. Bell, C.J. *et al*. The Blancan, Irvingtonian and Rancholabrean mammal ages. In *Late Cretaceous and Cenozoic Mammals of North America: Biostratigraphy and Geochronology* (ed. Woodburne, M.O.) 376 pp. (Columbia University Press, New York, 2004).

81. Flynn, J.J. Geochronology of Hemphillian-Blancan aged strata, Guanajuato, Mexico, and Implications for timing of the Great American Biotic Interchange. *J. Geol.* **113**, 287-307 (2005).
